# Skeleton-based motion prediction: A survey

**DOI:** 10.3389/fncom.2022.1051222

**Published:** 2022-10-28

**Authors:** Muhammad Usman, Jianqi Zhong

**Affiliations:** College of Electronics and Information Communication Engineering, Shenzhen University, Shenzhen, China

**Keywords:** skeleton-based motion prediction, survey, human motion prediction, 3D human pose representation, deep learning

## Abstract

Human motion prediction based on 3D skeleton data is an active research topic in computer vision and multimedia analysis, which involves many disciplines, such as image processing, pattern recognition, and artificial intelligence. As an effective representation of human motion, human 3D skeleton data is favored by researchers because it provide resistant to light effects, scene changes, etc. earlier studies on human motion prediction focuses mainly on RBG data-based techniques. In recent years, researchers have proposed the fusion of human skeleton data and depth learning methods for human motion prediction and achieved good results. We first introduced human motion prediction research background and significance in this survey. We then summarized the latest deep learning-based techniques for predicting human motion in recent years. Finally, a detailed paper review and future development discussion are provided.

## 1. Introduction

**Figure 1:**
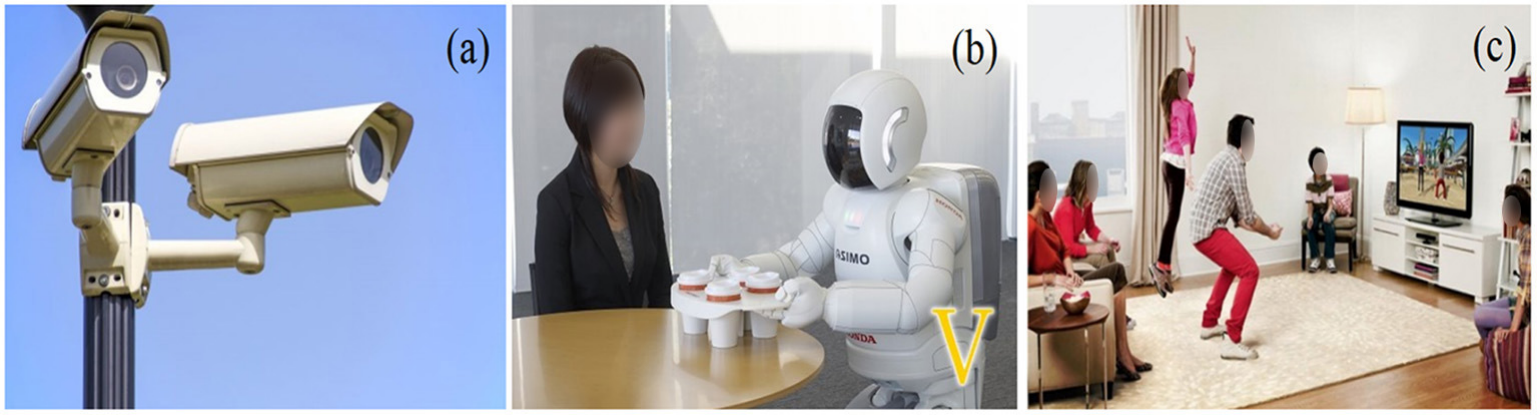
HMP applications: **(a)** Surveillance cameras. **(b)** Human-Robot Interaction. **(c)** Home entertainment.

Humans can predict and make accurate short-term predictions about the world around them based on previous events. In the field of virtual reality, human-computer interaction is an important research direction. How to make the machine able to imitate the human's ability to make corresponding predictions on the actions of the human body is a research hotspot in this field for computers. Predicting human motion is vital for timely human-robot handover, obstacle avoidance, and person tracking. Although a simple physical phenomenon. For example, the motion of inanimate objects can be predicted by the known laws of physics. But there is no simple equation governing a person's conscious movement. It is challenging to solve everyday problems, such as predicting what actions an individual will take next in the physical environment. It is due to the fact that the state of various parts of the human body can be in many possible permutations and combinations. But one can still predict actions in the next life by decomposing them into distinct categories or states and inferring their dynamic consequences to help computers perceive the movement trend of the human body in advance. Human behavior modeling is a classic problem. The human body is modeled by obtaining certain information to achieve the purpose of human behavior prediction. This kind of research is a relatively new research point. Today, deep learning is gradually being applied to a wider range of fields. The machine's understanding of the environment can be more in-depth and proficient in various ways, such as human motion prediction, have several applications as described in Figure 1. Machines have not yet been able to understand and predict human movements as well as humans. There is much room for progress in studying human movement prediction in such an environment. At the same time, existing research uses BNN to analyze the action sequence and generate subsequent action frames, such as the ERD model (Fragkiadaki et al., 2015a) model, S-RNN (Jain et al., 2016) model, and so on. However, in these studies, the length comparison of the action sequences generated does not exceed one second, and some actions shake correspondingly. Hence, it is impossible to make good predictions for long-term actions. Therefore, designing a neural network model more aligned with human kinematics to predict a complete set of actions is of great significance in human-computer interaction, robot choreography, and action recognition.

Existing methods for predicting human actions are mainly divided into video methods and human skeleton motion data methods. Existing video generation methods aim to accomplish two tasks; the first is video prediction (Oh et al., 2015), that is, the model needs to learn the motion pattern from a series of observation frames and predict the next frame. These methods are usually based on recurrent neural networks. Recurrent Neural Networks (RNNs) have an excellent ability to model continuous data; they usually only achieve good results in short-term predictions that are predictable when the thinking is simple and quiet. However, long-term prediction results, such as blurring and object deformation, generally suffer from low image quality. The second method uses human skeleton information directly for human action prediction. These methods use generative neural network models to generate spatiotemporal maps or variational autoencoders (Kingma and Welling, 2013) to predict dense trajectories of pixels. However, suppose the generated sequence frame does not have certain geometric constraints. In that case, the objects in the scene are very arbitrary and move irregularly, which will cause the generated objects to be very different from the originally required targets. A common limitation of both types of methods is that the joint structures of moving objects in the sequence frames of the previous part are not well modeled in generative models. Since previous generative methods only take the entire appearance as input, it is difficult for the model to understand the structural relationship between joints without supervision. As a result, a large deformation occurs during the movement process (Pan and Liu, 2008; Pan et al., 2013; Pan, 2015), and the quality of the generated video is far from satisfactory. The use of human skeleton motion data can well extract motion information. By directly learning the motion information of bones, unnecessary reconstruction of human body shape and video background can be effectively avoided, and limited information can be effectively used to achieve accurate learning of the purpose of human action.

**Figure 2:**
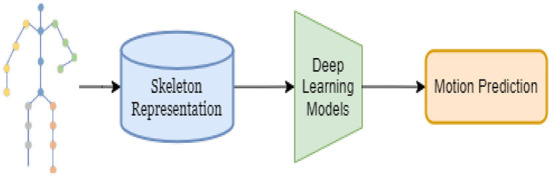
The overall foundation for deep learning methods for skeleton-based motion prediction.

3D skeleton-based HMP aims to forecasts future poses given a history of their previous motions based on human skeletons. This research has been applied in many practical application scenarios such as human-computer interaction (Koppula and Saxena, 2013, 2015), pedestrian tracking, (Alahi et al., 2016; Bhattacharyya et al., 2018), autonomous driving (Huang and Kitani, 2014; Chen et al., 2021), and animal tracking (Fragkiadaki et al., 2015a). Human motion animation (Hodgins, 1998), motion analysis and biomechanical analysis in sports have grown alongside computational and video graphic technology. Gross movement analysis, cellular and molecular elements of healing in relation to stress and strain, and cardiovascular or respiratory system mechanics are biomechanical applications in human movement (Zheng and Barrentine, 2000). The goal of virtual reality research is to build a simulated virtual environment so users can interact with items to obtain a “immersive” effect, like the actual world (Zhao et al., 2019). Animations from human body motion using direct and inverse kinematics (Sanna et al., 2015). Biomechanical analysis of gait data (Abu-Faraj et al., 2015) includes ground response forces, plantar pressures, kinematics, kinetics, dynamic electromyography, and energy consumption. Over the past several decades, traditional works employed Gaussian processes (Wang, 2005), Markov models (Taylor et al., 2006; Lehrmann et al., 2014a,b), linear dynamic systems (Vladimir et al., 2000), and Boltzmann Machine (Schlkopf et al., 2007) to capture human motion dependencies. In recent years, with the continuous development of deep learning methods in most existing computer vision tasks, deep learning-based methods show surprising performance in HMP. Deep learning structure can capture hierarchical dependencies of human motion for impressive prediction performance. We are showing in Figure 2. The overall foundation for deep learning methods for skeleton-based motion prediction.

In the following, we will introduce the two main deep learning-based methods: (1) RNN-based methods; (2) GCN-based methods, which is the main methods for skeleton-based motion prediction recently.

## 2. Deep learning-based HMP with 3D skeleton

### 2.1. RNN-based methods

RNN is a continuously rented neural network used in neural network model in which neural activations are processed when connections are made. They are looped through the network test success and generated motion prediction. A predictive RNN is applied by predicting visually localized numbers into a vocabulary of visual words. And the distribution of the next word in the order of the visual vocabulary given the visual at a specific position. Therefore, RNN can also be used in the study of human action prediction. Due to the sequential characteristics of human motion, Recurrent Neural Networks (RNNs)-based methods.

Recurrent neural networks (RNNs), which can handle sequential data with variable lengths, have recently been developed (Graves et al., 2013; Sutskever et al., 2014), Having demonstrated their proficiency in language modeling (Mikolov et al., 2011), video assessment (Li et al., 2016; Ma et al., 2016; Ni et al., 2016), and motion prediction based on RGB (Wu et al., 2015), At the one hand, the use of these networks in motion prediction using skeletons has also demonstrated promising but relatively limiting results (Shahroudy et al., 2016; Li et al., 2018). In independently recurrent neural network (IndRNN; Li et al., 2018), they regulated gradient back-propagation over time to prevent gradient vanishing and exploding. The independence and interlayer connections of neurons in the same layer account for their behavior. Intra-frame joint spatial representation and inter-frame time representation are significant aspects of this difficulty. Because they only considered temporal information and neglected spatial joint interdependence. Liu et al. (2018) suggested a network with spatiotemporal LSTM (STLSTM) adding another trust gate to simulate temporal and spatial dynamics and dependencies. The kinematic link between body joints was used to create a traversal technique, which was then used to better model spatial interdependence. The trust gate can determine when and how to update, delete, or remember internal memory's long-term context. The offered methods model only long-term dependence, not short-term or medium-term, and depend on relative coordinate systems that depend on specific joints (Defferrard et al., 2016). Employing the Gram Schmidt method, they converted a human skeleton into the brain's cognitive coordinate system. Instead of using the raw skeletons, they collected prominent motion elements from the transformed skeletons.We are comparing deep network framework techniques in Table 1.

**Table 1:** The comparison of deep network framework methods.

RNN-based methods	Skeleton sequences, which may be thought of as a time series of joint coordinate locations for RNN-based algorithms can be processed as time series data because of the RNN's particular structure. Despite producing good results, RNN-based algorithms are unable to effectively learn the spatial relationships between skeletal joints.
GCN-based methods	The joints serve as the corner points and the edges of the skeleton's naturally occurring graph, which is arranged in a non-Euclidean space. The skeleton data's graph structure cannot be utilized by the prior approaches, and they are difficult to apply to skeletons with arbitrary forms. A GCN-based model is created on top of a series of skeleton graphs, allowing it to fully investigate the discriminative data in both the spatial and temporal domains.

### 2.2. GCN-based methods

However, the motion prediction performance of these RNNs-based methods suffer from training difficulty (Pascanu et al., 2013) and error accumulation (Fragkiadaki et al., 2015b; Martinez et al., 2017), leading to unsatisfactory motion predictions, especially in long-term prediction. To solve these problems in RNNs-based methods, some researchers tried to make use of Graph Convolution Networks (GCNs)-based methods. In Mao et al. (2019), a human stance was represented as a graph structure that connected every joint that was close by. In addition, they suggested a new GCN that would connect the graph automatically rather than manually. Next, a novel graph network was suggested for use as a generator in GANs (Cui et al., 2020). Additionally, a dynamic learning graph was employed, but it wasn't the same as a standard one because it can connect joints that are geometrically separated but only explicitly learn pairings of natural joints. Li et al. (2020) created a unique GCN called DMGNN that included a dynamic multi-scale graph to describe the anatomy of the human skeleton. The internal relationships of the human body can be completely modeled using the multi-scale graph. Additionally, it can be applied to dynamic learning across network levels. A proposed graph-based gate recurrent unit was used for this assignment to create future poses. A unique multi-task graph convolutional network (MT-GCN) with a shared context encoder was also proposed by Cui and Sun (2021) to produce high-fidelity HMPs from shared context encoder (SCE). The faulty pose was repaired using both graph structure and a temporal self-attention technique that chose the most pertinent information from the entire sequence. The correlation of body parts was also captured in Zhou et al. (2021) using a similar multi-scaled approach.

## 3. Datasets

In many areas of algorithm development, datasets are essential. They typically serve a key role in facilitating network learning and measuring performance as a common ground. In addition, the field has become more significant and complex due to the increased quality of datasets. Recent years have seen a significant increase in interest in deep learning, which is useful in part because to the enormous amount of data. As a result, new datasets are being produced to solve the problems. To enhance learning, only a few human motion prediction datasets are used. There are just a handful datasets used for HMP to improve learning. we are showing over dataset comparison in Table 2.

**Table 2:** Dataset comparison.

**Dataset**	**Sensors**	**Number of joints**	**FPS**	**Location**	**Year**
H36M	10 Vicon T40	32	25	Indoor	2014
CMU	12 infrared cameras	38	25	Indoor	2003
3DPW	A Hand-held Smartphone Camera	17	30	Outdoor	2018

### 3.1. Human 3.6M (H36M)

The H36M public dataset captures information on human motion, including five female and six male 3D human poses and related photos. It includes all 3.6 million data points gathered from 4 separate Vicon motion capture system views. These postures feature 15 difficult action situations, such as giving directions, conversing, eating, greeting, making a phone call, posing, shopping, waiting, smoking, taking pictures, walking together, and running with a dog. Asymmetries such as strolling with a hand in a pocket or carrying a bag on the shoulder are also present in each scenario. Thirty-two skeleton joints make up an entire skeleton, and pose parameterizations comprise skeleton representations of joint positions and joint angles. Researchers always divided these poses into seven distinct individuals (S1, S5, S6, S7, S8, S11), removed duplicate points from the human stance, and retrained 25 points in these studies. Using down-sampling, 25 frames per second is set (FPS). Datasets are openly accessible at https://vision.imar.ro/human3.6m.

### 3.2. Mocap CMU

Twelve infrared cameras at Carnegie Mellon University captured data made available to the public in 2003. The human body has markers affixed on it. There are 144 different subjects in this dataset, including window washing, basketball, traffic control, jumping, jogging, and soccer. There are 38 joints in the parameterized human posture. In the experiments, these samples are frequently split into training and test sets. The segments are down-sampled in order to obtain the 25 fps frame rate. This data set has been made available to the public at https://mocap.cs.cmu.edu/.

### 3.3. 3DPW

The 3DPW dataset is mainly given for situations in nature. It is a sizable dataset that is openly available and contains more than 51,000 indoor and outdoor postures in addition to 60 film clips. Using an IMU or a hand-held smartphone camera, this dataset was recorded. The IMU is typically used by two actors to carry out a variety of actions, including shopping, exercising, hugging, conversing, taking selfies, riding the bus, playing the guitar, and relaxation. There was a total of seven actors wearing 18 different outfits. There are 17 joints used in each position. It is 30 frames per second. Dataset are openly accessible.

## 4. Discussion and future work

Skeleton-based motion prediction has grown in popularity and useful as a computer vision task during the past few years. Deep learning techniques and skeleton data are strong and useful tools in this field that significantly advance research. This advancement is credited with the expressiveness of skeleton data, model's adaptability, and training method's high effectiveness. The following are the significant contributions: (1) we provide a thorough analysis and summary of current best practices for 3D skeleton motion prediction using deep learning approaches, including the most recent algorithms used in RNN-based and GCN-based methods. Then, using deep learning and data from 3D skeletons, we describe a general framework for motion prediction techniques; (2) To the best of our knowledge, this is the first work that combines the analysis of the GCN method's multiple evolutionary approaches with the research based on those methods. One of the difficulties in skeleton-based human motion prediction is the wide range of perspectives in the recorded human action data. The two causes of this issue are the camera placement and how people move. Additional issues include making the most of joint interdependence, optimizing the spatial-temporal graph, and effectively utilizing bone information. Researchers are still dealing with these difficulties, and they will need to be researched and resolved in the future. Future research areas worth looking at include occlusion and self-occlusion, lightweight models, applications on mobile devices, and multi-task learning. Furthermore, another intriguing area worth investigating is the interpretability of motion prediction models. We weigh the benefits and drawbacks of the various techniques. Several potential study routes are discussed in light of the survey's findings, highlighting the wide range of opportunities in the subject despite its current level of development. Future research should focus more on significant and complex datasets.

## Author contributions

MU: methodology and software. MU and JZ: formal analysis, investigation, writing—original draft preparation, writing—review and editing, and supervision. All authors have read and agreed to the published version of the manuscript.

## Conflict of interest

The authors declare that the research was conducted in the absence of any commercial or financial relationships that could be construed as a potential conflict of interest.

## Publisher's note

All claims expressed in this article are solely those of the authors and do not necessarily represent those of their affiliated organizations, or those of the publisher, the editors and the reviewers. Any product that may be evaluated in this article, or claim that may be made by its manufacturer, is not guaranteed or endorsed by the publisher.
